# CSDE1 depletion inhibits tumor progression through enhancing B-cell infiltration in NSCLC

**DOI:** 10.1038/s41419-025-08282-9

**Published:** 2025-12-06

**Authors:** Wanting Li, Shijian Xiang, Ke Liu, Yang Wang, Qiuqi Li, Qianying OuYang, Yan Zhan, Bing Yu, Hui Chen, Bingchen Ge, Jiajia Cui, Jiye Yin, Aoxiang Guo

**Affiliations:** 1 Department of Clinical Pharmacology, Xiangya Hospital, Central South University, Changsha, Hunan PR China; 2 National Clinical Research Center for Geriatric Disorders, Xiangya Hospital, Central South University, Changsha, Hunan PR China; 3 Engineering Research Center of Applied Technology of Pharmacogenomics, Ministry of Education, Changsha, Hunan PR China; 4 Guangxi Key Laboratory of Tumor Immunology and Microenvironmental Regulation, Guilin Medical University, Guilin, PR China; 5 Guangxi Health Commission Key Laboratory of Tumor Immunology and Receptor-Targeted Drug Basic Research, Guilin Medical University, Guilin, PR China; 6 Department of Pharmacy, The Seventh Affiliated Hospital, Sun Yat-sen University, Shenzhen, PR China; 7 Shenzhen Key Laboratory of Chinese Medicine Active Substance Screening and Translational Research, the Seventh Affiliated Hospital, Sun Yat-sen University, Shenzhen, PR China; 8 Department of Pharmacy, Xiangya Hospital, Central South University, Changsha, Hunan PR China; 9 Department of Pharmacy, Union Hospital, Tongji Medical College, Huazhong University of Science & Technology (HUST), Wuhan, Hubei PR China; 10 Department of Lymphoma & Hematology, Hunan Cancer Hospital, the Affiliated Cancer Hospital of Xiangya School of Medicine, Central South University, Changsha, PR China; 11 Department of Pharmacy, Shanghai Children’s Medical Center, School of Medicine, Shanghai Jiao Tong University, Shanghai, PR China

**Keywords:** Cancer microenvironment, Tumour immunology

## Abstract

Although most immunotherapy research focuses on T cells, increasing evidence highlights the significant role of tumor-infiltrating B lymphocytes (TIL-Bs) in cancer therapy. CSDE1 has been implicated in various cancers and immune responses. This study investigates the role of CSDE1 in lung cancer progression and its impact on the tumor immune microenvironment. We found that CSDE1 promotes lung cancer progression in vivo but not in vitro. Using the tumor-bearing mice model with *Csde1* knockout, we demonstrated that *Csde1* deletion significantly inhibited tumor growth. Single-cell RNA sequencing revealed that *Csde1* knockout reshaped the tumor immune microenvironment, particularly by significantly increasing TIL-B levels, a finding confirmed by flow cytometry and immunofluorescence. Moreover, *Csde1* knockout enhanced B-cell-mediated humoral immunity. Notably, depleting B cells in *Csde1* knockout mice reversed the inhibitory effect of *Csde1* deletion on tumor progression, underscoring the critical role of B cells in this process. These findings suggest that CSDE1 facilitates tumor progression by modulating TIL-Bs and the broader immune microenvironment. This study provides a new potential target and valuable insights into tumor immunotherapy, emphasizing the importance of B cells in cancer treatment strategies.

## Introduction

Cold shock domain-containing E1 (CSDE1), also known as upstream of N-RAS (UNR), is an RNA-binding protein characterized by nine cold shock domains [1]. It serves as a key regulator of translational reprogramming and participates in diverse biological processes, including cell cycle regulation, apoptosis, and differentiation [2–4]. Recent studies have identified CSDE1 as a potential prognostic biomarker and therapeutic target in cancer [3–6]. Emerging evidence also suggests that CSDE1 is involved in the regulation of tumor-immune interactions. For example, Jiadi et al. demonstrated that CSDE1 modulates immune recognition during tumorigenesis and may serve as a predictive marker for cancer immunotherapy efficacy [7]. Additionally, a point mutation in CSDE1 was shown to create a neoepitope recognized by nontolerized T cells, facilitating tumor immune evasion from VSV-IFNβ during hemolysis [8]. Importantly, CSDE1 plays a context-dependent role in cancer, functioning as an oncogene in malignancies such as melanoma while acting as a tumor suppressor in other cancer types [2]. However, the specific role of CSDE1 in lung cancer and its underlying mechanisms in tumor progression and immune regulation remain insufficiently understood.

Lung cancer remains the leading cause of cancer-related mortality worldwide and is the second most commonly diagnosed malignancy [9, 10]. Non-small-cell lung cancer (NSCLC), including lung adenocarcinoma (LUAD) and lung squamous cell carcinoma (LUSC), accounts for approximately 85% of all lung cancer cases [11]. Traditional treatment modalities have shown limited long-term efficacy. Over the past decade, significant advancements have been achieved through targeted therapies and immunotherapies, such as immune checkpoint inhibitors, which are tailored to genetic and molecular characteristics [11, 12].

Immunotherapy which reshapes the tumor immune microenvironment (TIME) eliminates tumor cells through the tumor immune response [13, 14]. Tumor cells, immune cells, cytokines, and other factors constitute the TIME. The interaction between tumor cells and immune cells determines tumor progression by regulating the balance between pro- and antitumor inflammatory mediators [15, 16]. Tumor-infiltrating immune cells, such as tumor-infiltrating T lymphocytes and tumor-infiltrating B lymphocytes (TIL-Bs), play important roles in the TIME [17]. Extensive studies suggest that T cells are the main executors of antitumor immunity. Currently, most immunotherapy strategies target antibodies or small molecules on T cells to alter the function of T cells and achieve therapeutic effects, such as ICB antibodies against CTLA-4 and PD-1 [18, 19]. However, the TIME centered around T cells is unable to fully explain the complexity of human cancer. Recently, an increasing number of studies have reported that TIL-Bs, which include B cells and plasma cells, have strong prognostic significance and the efficacy of tumor therapy in various types of tumors [20–22]. TIL-Bs can shape T-cell responses and secrete antibodies, cytokines and chemokines, which contribute to antitumor immunity.

In this study, we investigated the role of CSDE1 in the NSCLC TIME. Our findings reveal that CSDE1 depletion significantly suppresses tumor progression by reshaping the TIME in a B-cell-dependent manner.

## Materials and methods

### Cell culture

H1299, A549 and Lewis lung cancer (LLC) cell lines were purchased from the National Collection of Authenticated Cell Cultures. They were routinely cultured in RPMI-1640 medium supplemented with 10% fetal bovine serum (Umedium, He Fei, China) at 37 °C in humidified air with 5% CO_2_.

### Animal experiments

BALB/c-Nu athymic nude mice, *Csde1*^*flox/flox*^ (NC) C57BL/6 and *Ubc-Cre*^*ERT2*^ C57BL/6 mice were purchased from Gempharmatech Co., Ltd. (Nanjing, Jiangsu, China). *Csde1*^*flox/flox*^ mice were crossed with *Ubc-Cre*^*ERT2*^ mice to obtain homozygous *Csde1*^*flox/flox*^*Ubc-Cre*^*ERT2*^ (CKO) C57BL/6 mice. All the mice were housed in a specific pathogen-free environment, maintained in a standard temperature- and humidity-controlled environment on a 12-h light/dark cycle, and provided sterilized water and standard rodent food. The experiments were performed during the photoperiod. The experiments were approved by the Institutional Animal Care and Use Committee (IACUC), Sun Yat-Sen University (Approval no. SYSU-IACUC-2022-000750).

For the human-origin cell-derived xenograft (CDX) lung cancer model, 6–8-week-old male BALB/c-Nu athymic nude mice were subcutaneously inoculated with 8 × 10^6^ H1299 cells, with or without CSDE1 knockdown (shCSDE1). The body weights of the mice and the tumor volumes were measured every 2 days until the tumor diameter reached 1 cm.

For the mouse-derived CDX lung cancer model, 6–8-week-old CKO and NC male C57BL/6 mice were subcutaneously inoculated with 5 × 10^6^ LLC cells. Tamoxifen was injected intraperitoneally to induce systemic knockout of *Csde1*. The body weights of the mice and the tumor volumes were measured every 2 days until the tumors grew to the size of a mung bean.

### Genotyping

The toes of newborn mice within 10 days were collected for genotype identification with a Mouse Direct PCR Kit (catalog: B40015; Bimake). The primers used are listed in Table 1.

**Table 1:** The primers for PCR.

Gene	Primer
*Csde1*-Flox	Primer-F: 5’ - CATGGGTGCTAGGTATTGATC - 3’
*Csde1*-Flox	Primer-R: 5’ - CTCTGTGCAACTATGGCTTG - 3’
UBC-Cre	Primer-F: 5’ - AGCGATGGATTTCCGTCTCTGG - 3’
UBC-Cre	Primer-R: 5’ - AGCTTGCATGATCTCCGGTATTGAA - 3’

### Bone marrow smear analysis

The mice were euthanized, bone marrow cells were extracted, and the cells were resuspended in PBS. The resulting bone marrow suspension was spread onto glass slides. After air drying, the suspension was fixed via Wright’s stain. Equal volumes of ultrapure water were then added and mixed. The slides were washed on a shaker, dried in a 37 °C incubator, and mounted with neutral resin before being examined under a microscope.

### H&E staining, immunohistochemistry (IHC) and multiplex immunohistochemistry (mIHC) analyses

Mouse tumor tissues were fixed in 4% paraformaldehyde for 24 h. After dehydration, the tissues were embedded in paraffin, sectioned at a thickness of 4 μm, and stained with hematoxylin and eosin (H&E) for histological evaluation under a microscope.

For IHC and mIHC, prepared paraffin sections were placed in a 65 °C drying oven for 2 h. The sections were subsequently deparaffinized and rehydrated through a series of solutions, including xylene and graded ethanol (100, 95, and 75%). Antigen retrieval was performed via the use of citrate buffer solution under high-temperature heating. For IHC staining, the primary antibody used was anti-PCNA (ab19166, Abcam), anti-Ki67 (ab16667, Abcam), and anti-CD19(ab245235, Abcam).

For multiplex immunohistochemistry (IHC), the sections were stained with a 4-color Fluorescence kit (AFIHC035, Aifang Biotechnology) according to the manufacturer’s instructions. The primary antibodies applied included CD19 (AFRM0354, Aifang Biotechnology), CD3 (AFRM0030, Aifang Biotechnology), CD4 (AFRM0003, Aifang Biotechnology), CD8 (AFRM0004, Aifang Biotechnology), CSDE1 (ab201688, Abcam), and CD21(AFRM0156, Aifang Biotechnology). The fluorescence signal was captured by KFBIO KF-FL-020.

### Western blot

Cells or tissues were lysed, and the proteins were extracted with radioimmunoprecipitation assay lysis buffer. The proteins were subsequently separated via SDS‒PAGE and transferred to a polyvinylidene fluoride membrane (Millipore, 10600023, MA, USA). The membrane was incubated with a specific primary antibody and then incubated with a secondary antibody. The antibodies used were: CSDE1 (ab201688, Abcam), GAPDH (G8795, Sigma) and β-Actin (A5441, Sigma). Finally, the protein expression levels were detected and visualized with an enhanced chemiluminescence (ECL) developer (RPN2232, GE Healthcare, USA).

### Flow cytometry

The collected spleen and tumor tissues were minced and ground to prepare single-cell suspensions. Each sample was blocked with anti-mouse CD16/CD32 (156603, BioLegend) and incubated with Zombie Aqua for viability staining, followed by incubation with fluorescent antibodies, including F4/80 (123109, BioLegend), CD11c (25-0114-82, Invitrogen), CD8 (561109, BD), NK-1.1 (108731, BioLegend), CD45R (103229, BioLegend), CD4 (100429, BioLegend), CD45 (103115, BioLegend), CD3 (100231, BioLegend) and CD11b (101241, BioLegend). The samples were subsequently incubated with diluted lysis buffer to lyse red blood cells. After centrifugation, the supernatant was discarded, and the cells were resuspended in PBS for further analysis.

### Enzyme-linked immunosorbent assay (ELISA) analysis

Tumor tissues were homogenized in cold PBS using an automatic fast low-temperature grinder. The supernatants were subsequently collected after centrifugation and measured via different ELISA kits (Cat. numbers: CSB-E04661m; CSB-E04569m; CSB-E04741m; CSB-E04627m; CSB-E04594m; Cusabio) according to the manufacturer’s instructions.

### scRNA sequencing and data analysis

The mice were euthanized to collect fresh tumor tissue, which was processed into single-cell suspensions via a mouse tumor dissociation kit (Miltenyi Biotec, Germany). Single-cell RNA sequencing was conducted following the manufacturer’s protocol, utilizing the DNBelab C4 Single-Cell 3’ RNA-Seq Library Chemistry and DNBelab C4 V2 Microfluidic Chip (MGI Tech, Shenzhen, China). Libraries were sequenced on the Illumina HiSeq2500 platform (Illumina, San Diego, CA, USA) and the DNBSEQ-T20×2 platform (MGI Tech, Shenzhen, China).

The sequence data were quantified and mapped to the *Mus musculus* reference genome (GRCm38) using scRNA_parse (version 1.0.1) and STAR software (version 2.7.2b). Single-cell sequencing analysis was performed via Seurat (version 4.0.5). Quality control steps were applied to exclude low-quality cells, which were defined as those with mitochondrial read percentages exceeding 5%, fewer than 200 detected genes, or more than 90% of the maximum detected gene count.

### B-cell clearance experiment

Tumor-bearing mice were intraperitoneally injected with tamoxifen for 5 consecutive days, followed by the intraperitoneal administration of an anti-mouse CD20 antibody (152104, BioLegend) to deplete B cells or IgG2b, κ Isotype Ctrl Antibody (400644, BioLegend). Subcutaneous tumor growth was subsequently monitored in the mice. Tumor tissues were collected at the endpoint, and flow cytometry was performed to assess B-cell counts in both the tumor and blood, confirming the efficiency of B-cell depletion.

### Statistical analysis

Statistical analysis of the data was performed via GraphPad Prism 8. The values are presented as the means ± standard errors of the means. Comparisons between two groups were conducted via an unpaired t-test. One-way ANOVA was used for multiple group comparisons. Statistical significance was defined as *P ≤ 0.05, ** *P* < 0.01, and *** *P* < 0.001. All independent experiments were repeated at least three times.

## Results

### CSDE1 promoted NSCLC progression in vivo but not in vitro

**Fig. 1:**
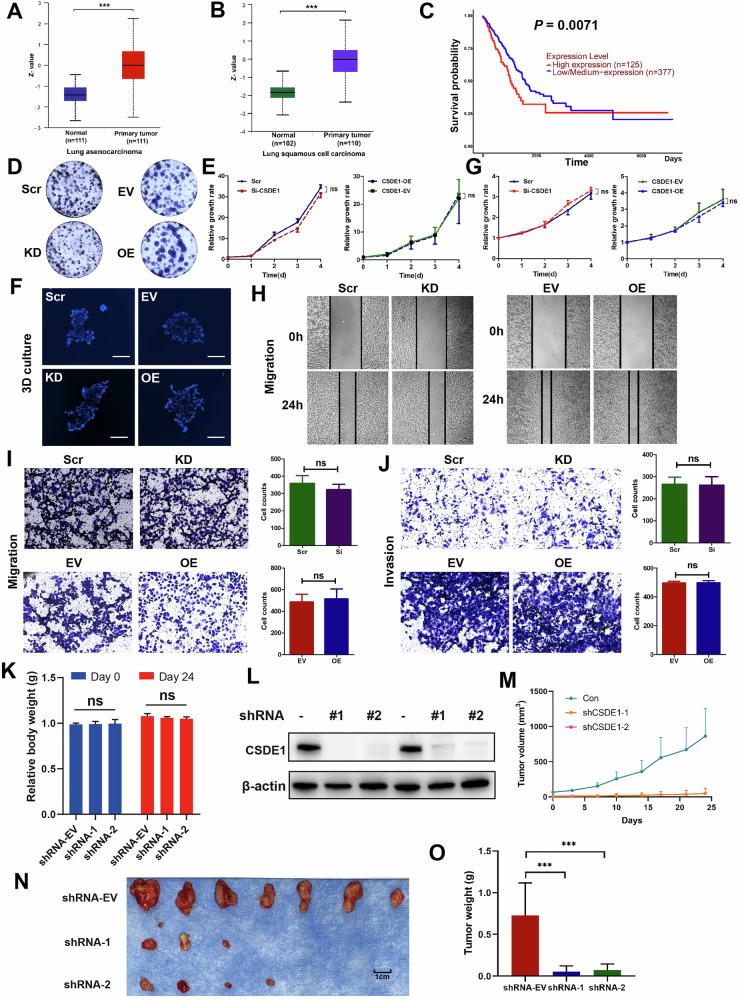
CSDE1 promoted tumor growth in nude mice but not in vitro. **A**, **B** Protein expression of CSDE1 in lung adenocarcinoma and lung squamous cell carcinoma based on the Clinical Proteomic Tumor Analysis Consortium (CPTAC). **C** Survival analysis of LUAD patients with high/low expression of CSDE1. *P* = 0.0071 **D** Colony formation assays of H1299 cells with high or low expression of CSDE1. **E** Proliferation of H1299 cells with high or low expression of CSDE1, as detected by a CCK8 assay. **F** Fluorescence image of H1299 cells cultured in matrix gel on the 4th day with high or low expression of CSDE1 stained with DAPI. **G** H1299 cell proliferation cultured in matrix gel with high or low expression of CSDE1. Wound healing (**H**), migration (**I**) and invasion (**J**) abilities of H1299 cells with high or low expression of CSDE1. **K** Relative body weight change (the present mouse weight divided by the initial weight) of the mice on day 0 and day 24 after transplantation of tumor cells (*n* = 7). **L** Knockdown efficiency of CSDE1 in tumors from nude mice transplanted with H1299 cells. CSDE1 was knocked down by using two shRNAs separately. **M** Tumor volume growth curve (mm^3^). **N** Representative morphological images of tumors. **O** Tumor weight distributions on Day 24. The data represent the mean ± SEM of three independent experiments, *P < 0.05, **P < 0.01, ***P < 0.001.

To evaluate the potential role of CSDE1 in NSCLC, we first analyzed the protein expression level of CSDE1 in patients with LUAD or LUSC on the basis of the Clinical Proteomic Tumor Analysis Consortium database and found that CSDE1 was more highly expressed in primary tumor tissues than in normal tissues (Fig. 1A, B). Survival analysis revealed that patients with lower expression of CSDE1 had a better prognosis (Fig. 1C). Next, we explored the role of CSDE1 in vitro and in vivo. The results revealed that both the knockdown (KD) and overexpression (OE) of CSDE1 in two NSCLC cell lines (H1299 and A549) had no significant effect on cell growth, migration or invasion (Fig. 1E–J and S1). A nude mouse model in which H1299 cells were transplanted with CSDE1-KD H1299 cells was subsequently constructed to explore the role of CSDE1 in tumor progression in vivo. We measured body weight and tumor volume over the following 24 days and found that there was no significant difference in mouse body weight gain between the two groups (Fig. 1K). On the 24th day, the mice were euthanized, and the subcutaneous tumors were removed. Western blot analysis confirmed that the expression of CSDE1 was downregulated by both shRNAs in tumors from CSDE1-KD mice (Fig. 1L and S8). Importantly, the tumor weight and volume growth rate in CSDE1-KD mice were much lower than those in control mice (Fig. 1M–O).

Taken together, these results demonstrated that CSDE1 expression does not affect tumor progression in vitro but contributes to tumor growth in vivo and is associated with prognosis in NSCLC patients.

### CSDE1 depletion in C57BL/6 mice significantly inhibited tumor progression

**Fig. 2:**
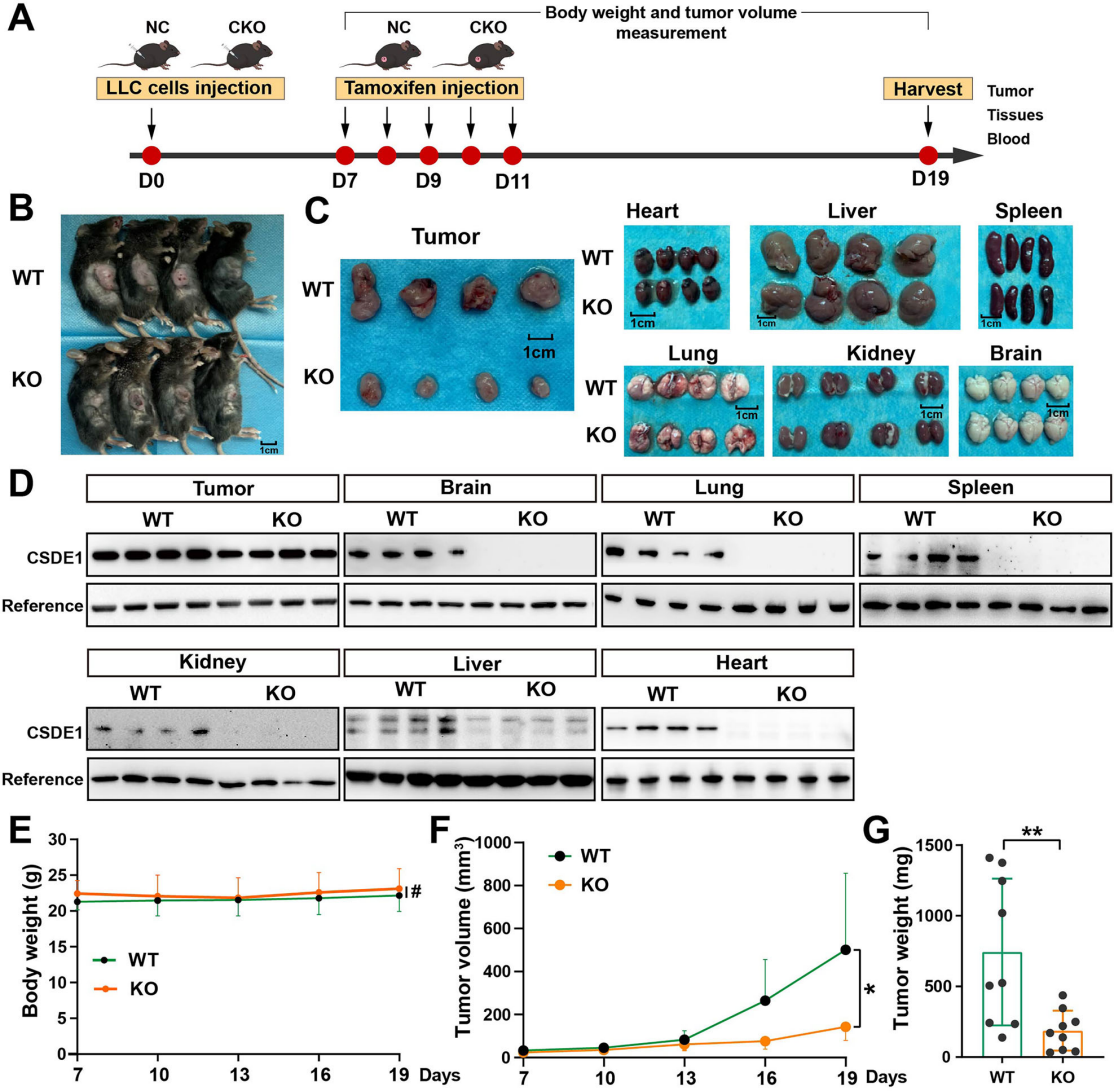
Tumor progression was significantly inhibited in *Csde1*-knockout C57BL/6 mice. **A** Treatment schema for constructing a tumor-bearing model in *Csde1*-knockout mice. **B** Representative morphological images of tumors (*n* = 4). **C** Representative morphological images of major mouse organs, including the heart, liver, spleen, lung, kidney, the brain. **D** Expression of CSDE1 in tumors and major organs, including the heart, liver, spleen, lung, kidney and brain. **E** Body weight change of mice from day 7 to day 19 (*n* = 9). **F** Tumor volume growth curve from day 7 to day 19 (*n* = 9). **G** Bar chart of tumor weight statistics on day 19.

To further explore the role and mechanism of CSDE1 in NSCLC, a *Csde1* systemic knockout (*Csde1*^*flox/flox*^*Ubc-Cre*^*ERT2*^, CKO) C57BL/6 mouse model was constructed. The mouse toe DNA was extracted for PCR to identify the genotype (Fig. S2). A CDX lung cancer model was subsequently constructed. As shown in Fig. 2A, the mice were euthanized on the 19th day, and the tumors and various organs were collected. As shown in Fig. 2B, the tumor volume was significantly lower in CSDE1 knockout mice than in control mice. No significant changes were observed in the other organs (Fig. 2C). Western blot analysis was performed to assess the protein expression of CSDE1 in various organs, including the heart, liver, spleen, lung, kidney, and brain, which confirmed the successful establishment of the systemic *Csde1* knockout mouse model (Figs. 2D and S10). No significant changes in body weight or a series of indicators, including body weight, physical appearance, organ-to-body weight ratios, routine blood parameters, serological markers of liver and kidney function, blood glucose, lipid levels, or serum electrolyte levels, were detected (Figs. 2E and S3, S4). The tumor growth volume and tumor weight were significantly lower in *Csde1*-knockout (KO) mice than in *Csde1*-wild-type (WT) mice (Fig. 2F, G).

### CSDE1 depletion induces histological alterations in mice

**Fig. 3:**
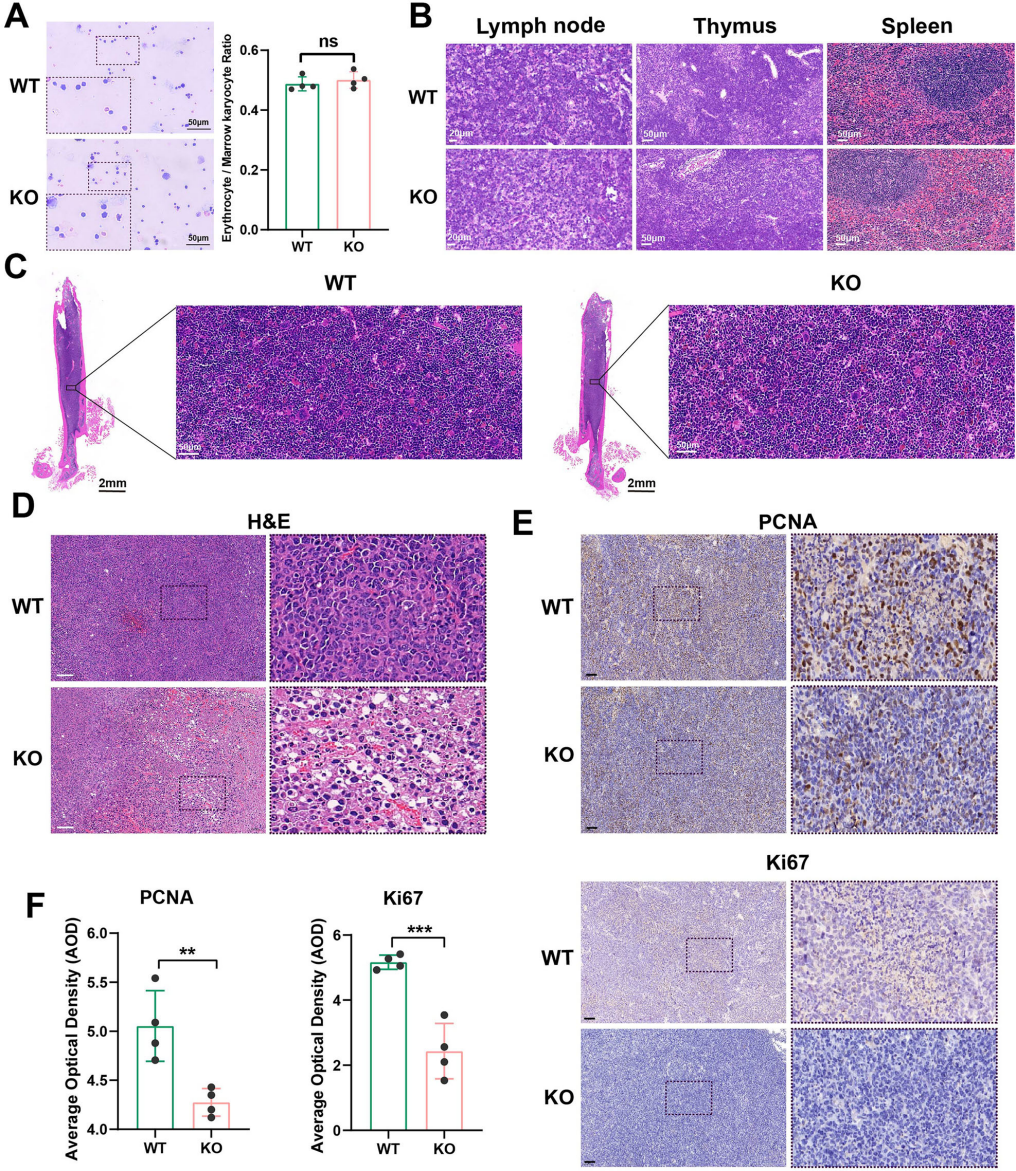
CSDE1 depletion induced the histological alterations in the mice. **A** Depiction of morphological characteristics and corresponding quantitative analysis derived from bone marrow smears. Scale bar: 50 μm **B** Histological presentation via HE staining of lymphoid tissues, including lymph nodes, thymus, and spleen. Scale bars: 20 μm (left), 50 μm (middle), 50 μm (right) **C** Histological visualization through HE staining of bone marrow samples. Scale bar: 50 μm **D** Histopathological illustration of tumor tissue following HE staining. Scale bar: 100 μm Immunohistochemical representation (**E**) alongside quantitative analysis (**F**) of the proliferative markers PCNA and Ki67 within tumor tissue sections. Scale bar: 100 μm.

We further analyzed the effects of *Csde1* knockout on histomorphology. Bone marrow smear analysis revealed no significant difference in the ratio of red blood cells to nucleated cells between *Csde1*-KO mice and *Csde1*-WT mice (Fig. 3A). No significant pathological changes were observed in the lymph nodes, thymus, or spleen (Fig. 3B). HE staining of the bone marrow also revealed no significant difference in the proportion distribution of bone marrow cells between the two groups (Fig. 3C). On the basis of these results, we preliminarily concluded that the general health condition of the model mice was good for subsequent studies.

HE staining of tumors revealed significant nuclear contraction and looser nuclear spacing in *Csde1*-KO mice and no significant histological damage in *Csde1*-WT mice, suggesting that knocking out *Csde1* has a significant effect on tumor growth (Fig. 3D). IHC of tumor tissue revealed that the level of the proliferative molecule PCNA in the tumor tissue of *Csde1*-KO mice was much lower than that in the tumor tissue of *Csde1*-WT mice (Fig. 3E), which suggested that systemically knocking out *Csde1* significantly inhibited the growth of NSCLC.

### Single-cell sequencing reveals CSDE1-mediated remodeling of the tumor immune microenvironment via B cells

**Fig. 4:**
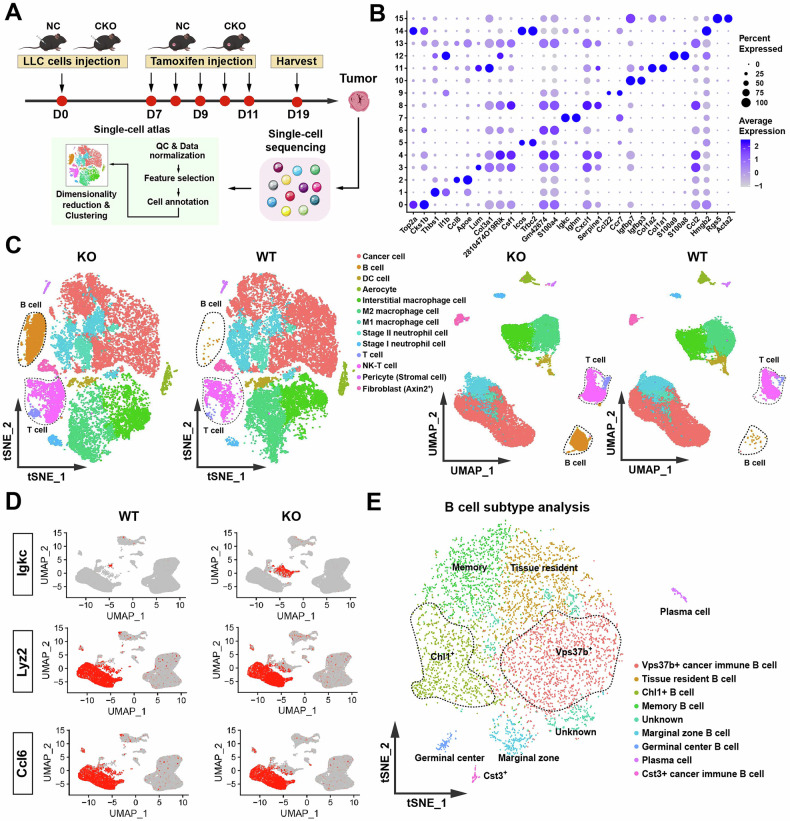
Single-cell RNA sequencing revealed CSDE1-mediated remodeling of the tumor immune microenvironment via B cells. **A** Single-cell RNA sequencing protocol design and procedure overview. **B** Bubble plot of marker gene expression in different clusters. **C** Identification of tumor-infiltrating immune cell subsets via the tSNE algorithm. **D** The three genes most significantly differentially expressed genes in B cells between the *Csde1*-WT and *Csde1*-KO groups. **E** Reclustering of **B** cells via the tSNE algorithm.

To elucidate the molecular mechanisms underlying *Csde1*-mediated tumor regulation, we conducted single-cell RNA sequencing (scRNA-seq) on dissociated tumor tissues from three biological replicates of *Csde1*-KO mice and three age/sex-matched *Csde1*-WT littermates (Fig. 4A). Postsequencing quality control (QC) was rigorously performed via Seurat (v5.2.1) with the following criteria: (1) exclusion of cells with <500 detected genes or >10% mitochondrial read content; (2) removal of potential doublets via Scrublet (threshold: doublet score >0.25); and (3) retention of genes expressed in ≥10 cells. All six samples exhibited comparable QC metrics (the average number of genes/cell was 3128, and the average mitochondrial gene proportion was 3.03%), confirming data homogeneity across groups (Fig. S5).

Unsupervised clustering through the UMAP (resolution = 0.8) and tSNE (perplexity = 30) algorithms resolved 16 transcriptionally distinct clusters (Fig. 4B and S6). Cell-type annotation via SingleR (v1.10) with the Mouse RNAseqData reference database, which was corroborated by canonical marker expression, identified 13 major immune and stromal subsets. Strikingly, comparative analysis revealed increased B lymphocyte infiltration in *Csde1*-KO tumors (Fig. 4C), suggesting that CSDE1 deficiency enhances B-cell recruitment or survival.

Focusing on the expanded B-cell compartment, further analysis revealed significant upregulation of Igk expression, a hallmark of mature B-cell differentiation, in the KO group (Fig. 4D). This transcriptional signature implied that CSDE1 might suppress terminal B-cell maturation in the tumor microenvironment. To dissect this heterogeneity, we performed subclustering, resolving nine functionally specialized subsets (Fig. 4E) as follows: memory B cells (Arid5a^+^Cd27^+^), marginal zone B cells (Cd1d1^+^Cr2^+^), germinal center B cells (Bcl6^+^Aicda^+^), plasma cells (Xbp1^+^Sdc1^+^), tissue-resident B cells (Itga1^+^Cd69^+^), and four previously uncharacterized populations designated by top markers: Chl1^+^ B cells (neuron interaction), Cst3^+^ cancer-immune B cells (antigen presentation), Vps37b^+^ cancer-immune B cells (vesicle trafficking), and Fam110a^+^ regulatory B cells. Quantitatively, four subsets dominated the landscape: memory B cells (28.6%), tissue-resident B cells (19.2%), Chl1^+^ B cells (15.1%), and Vps37b^+^ cancer-immune B cells (12.4%), collectively comprising 75.3% of the total B cells. This refined taxonomy not only corroborates established B-cell ontogeny but also reveals novel tumor-associated B-cell states potentially critical for antitumor immunity.

### Increased B lymphocyte infiltration was confirmed in tumors from *Csde1*-KO mice

**Fig. 5:**
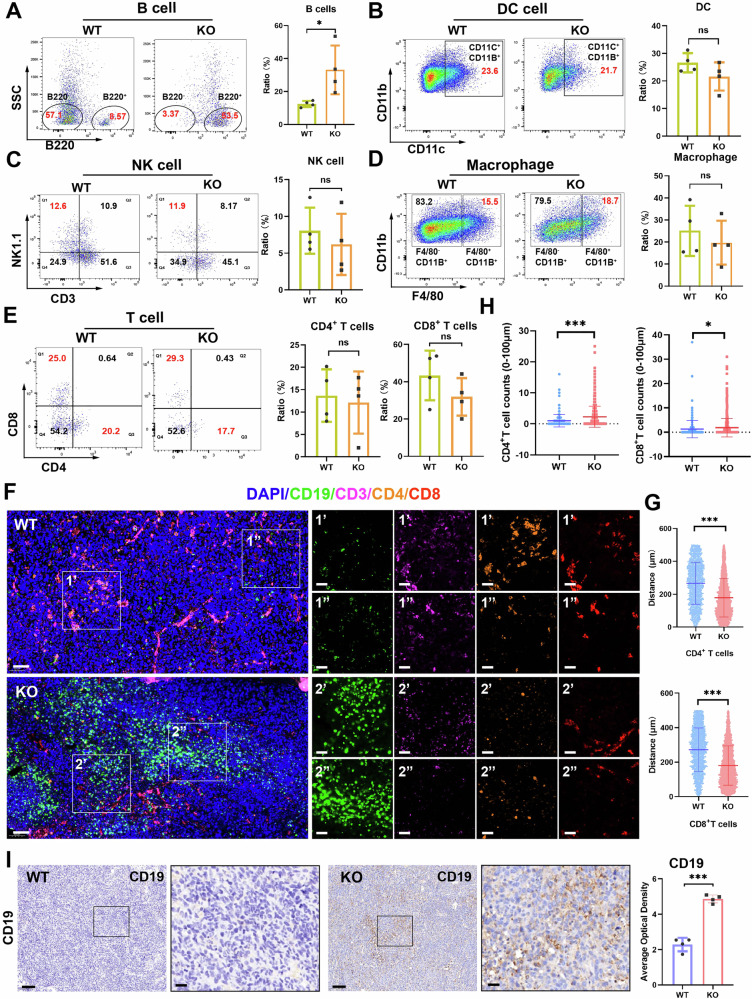
Increased B lymphocyte infiltration was confirmed in the tumors of *Csde1*-KO mice. **A**–**E** Flow cytometry analysis of B lymphocytes, DCs, NK cells, macrophages and T lymphocytes from tumor tissues. Representative images and quantification are shown (*n* = 4). **F** Multiplex immunohistochemistry analysis of tumor-infiltrating immune cells, including CD19^+^ B cells, CD3^+^ T cells, CD4^+^ T cells, and CD8^+^ T cells, from tumor tissue. Representative images are shown. **G** Distance from B cells to CD4^+^ T cells and CD8^+^ T cells within a range of 500 μm in tumor tissues from the *Csde1*-WT and *Csde1*-KO mice (*n* = 3). **H** Counts of CD4^+^ T cells or CD8^+^ T cells around B cells within a range of 100 μm in tumor tissues from the *Csde1*-WT and *Csde1*-KO mice (*n* = 3). **I** Immunohistochemical analysis of the CD19 level in tumor tissues. Representative images and quantification are shown (*n* = 4). The data represent the mean ± SEM of three independent experiments, *P < 0.05, **P < 0.01, ***P < 0.001.

To further confirm the results of single-cell sequencing, we first detected the level of tumor immune cell infiltration in the mice via flow cytometry. Compared with that in *Csde1*-WT mice, the level of B lymphocyte infiltration in the tumor tissues of *Csde1*-KO mice was significantly greater (Fig. 5A). However, there was no significant difference in the infiltration level of other immune cells, including macrophages, dendritic cells, natural killer cells, CD4^+^T cells, and CD8^+^ T cells (Fig. 5B–E). We further detected the level of B lymphocyte infiltration in tumor tissues via multiplex immunohistochemistry staining (mIHC) and IHC. These results indicated that the expression of the B-cell marker CD19 was significantly greater in *Csde1*-KO mice than in *Csde1*-WT mice, but the expression of the T-cell markers CD4 and CD8 was not significantly different between the two groups, which was consistent with the results of flow cytometry analysis (Fig. 5F–I). These results confirmed that knocking out *Csde1* could significantly increase the infiltration of B lymphocytes in the TIME. Further, we calculated the distance from B cells to CD4^+^/CD8^+^ T cells in tumor tissues. Both CD4^+^ and CD8^+^ T cells were closer to B cells within the radius of 500 μm in *Csde1*-KO mice than that in *Csde1*-WT mice (Fig. 5G). Besides, more CD4^+^/CD8^+^ T cells were observed around B cells within a close range of 100 μm in *Csde1*-KO mice (Fig. 5H). These results suggested that knocking out *Csde1* increased probability of spatial proximity between TIL-B cells with TIL-T cells.

Previous studies have demonstrated that tertiary lymphoid structures (TLSs) were associated with the adaptive immune response and positive prognosis in cancer. The significant feature of the TLS maturation stage is the appearance follicular dendritic cells (FDCs) networks and germinal centers (GC), with B cell regions surrounding the GC and T cell regions distributed around it [23, 24]. To investigate the role of CSDE1 in TLS maturation, we first examined H&E-stained sections to assess the presence and morphology of lymphoid aggregates (Fig. S7A). Following CSDE1 knockout, round and oval aggregates became evident within the tumor tissue. We then evaluated TLS maturity by mIHC, focusing on B- and T-cell compartmentalization and the abundance of (FDCs; CD21^+^) (Fig. S7B). The results showed that CSDE1 deletion led to incipient compartmentalization of B- and T-cell zones (B/T segregation) within the tumor, while FDCs were scarce, consistent with early-to-intermediate TLS maturation. No comparable structures were detected in the control group. These findings suggest that CSDE1 deficiency may, to some extent, promote TLS maturation. TLSs were few in number and exhibited limited maturation, likely because the development of fully mature TLSs requires sustained chronic inflammation, which is difficult to achieve in standard tumor-bearing mouse models. To further evaluate the potential role of CSDE1 in regulating TLS maturation, we reanalyzed the scRNA-seq data and examined TLS maturation–associated gene expression in B cells, FDCs, T cells, and endothelial cells. Most of these genes were expressed at higher levels in the KO group than in controls, suggesting a tendency for CSDE1 deletion to promote TLS maturation (Fig. S7C–F). In summary, CSDE1 deficiency can promote the formation and maturation of TLSs to some extent.

In addition, we wanted to further investigate whether *Csde1* could also increase the level of B lymphocytes or other immune cells in the immune organs of *Csde1*-KO mice. Considering that the spleen is the main site of specific immunity and the most abundant area of B cells, we detected the abundance of various immune cells in the spleen via flow cytometry. The results revealed no statistically significant differences in the proportions of various immune cells in the spleen, including macrophages, dendritic cells, NK cells, B lymphocytes, CD4^+^ T cells, and CD8^+^ T cells, between the *Csde1*-KO and *Csde1*-WT mice (Fig. S8). Taken together, these findings indicate that systemic knockout of *Csde1* specifically enhances B lymphocyte infiltration in the TME.

### CSDE1 depletion enhances B-cell-mediated humoral immunity

**Fig. 6:**
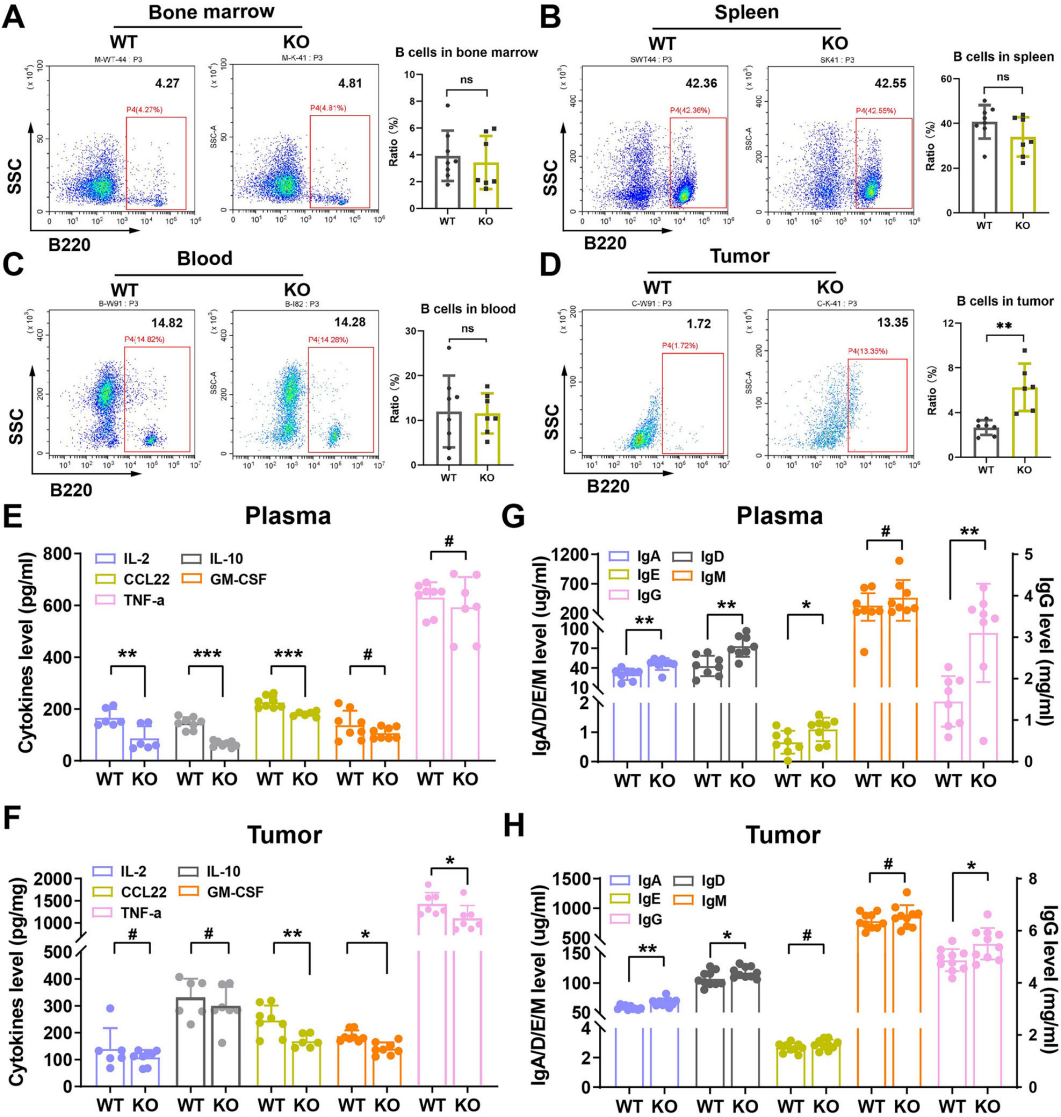
Knocking out *Csde1* changed the levels of cytokines and immunoglobulin. **A**–**D** Flow cytometry analysis of B lymphocyte cells in the bone marrow, spleen, blood and tumor tissues. Representative images and quantification are shown. Quantification of the levels of cytokines, including CCL22, GM-CSF, IL-2, IL-10 and TNF-α, in the plasma (**E**) and tumors (**F**). Quantification of immunoglobulin levels, including IgA, IgD, IgE, IgG and IgM, in plasma (**G**) and tumors (**H**). *P < 0.05, **P < 0.01,***P < 0.001, #: not significant.

B cells can recognize and present antigens, secrete antibodies, produce cytokines, generate immunological memory, and differentiate into plasma cells that produce immunoglobulin. B cells develop in the bone marrow, mature in the spleen, and eventually circulate in the blood. To explore the impact of CSDE1 on B-cell development, maturation and release, we first analyzed the levels of B lymphocytes in these tissues via flow cytometry and found no significant differences between *Csde1*-KO and *Csde1*-WT mice (Fig. 6A–D). Since cytokines play important roles in immune regulation, several representative cytokines, including CCL22, GM-CSF, IL-2, IL-10 and TNF-α, were detected via ELISA. Knocking out *Csde1* was found to significantly reduce CCL22, IL-2, and IL-10 levels in plasma, as well as CCL22, GM-CSF, and TNF-α levels in tumor tissue. (Fig. 6E, F). To further explore the effect of *Csde1* on B-cell function, we used ELISA to measure the level of immunoglobulin in plasma and tumor tissues and found that knocking out *Csde1* caused a significant increase in IgA, IgD and IgG in both plasma and tumor tissues (Fig. 6G, H). Therefore, knocking out *Csde1* might enhance the humoral immunity mediated by B cells.

### B-cell depletion reverses the impact of CSDE1 on tumor progression

**Fig. 7:**
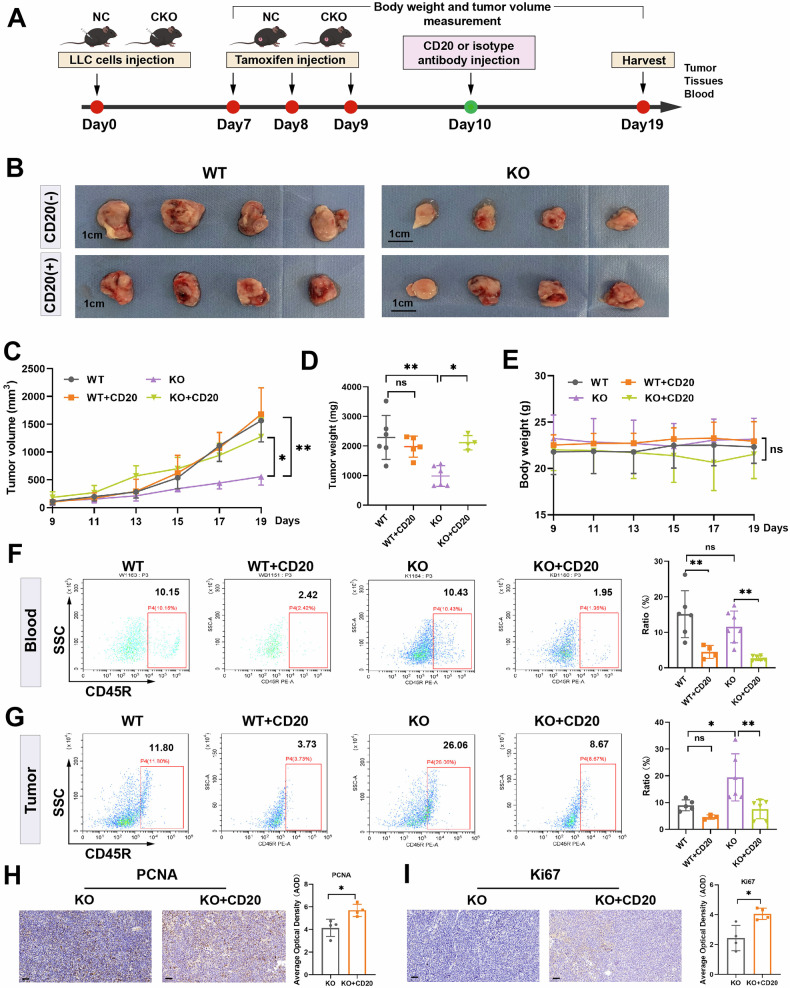
B-cell depletion reversed the inhibitory effect of *Csde1* knockout on tumor progression. **A** Treatment schema of the B-cell clearance experiment. **B** Tumor size on day 19 (*n* = 4). **C** Tumor volume growth curve from day 9 to day 19 (*n* = 4). **D** Tumor weights of the mice on day 19 (*n* = 4). **E** Changes in the relative body weights of the mice from day 9 to day 19 (*n* = 4). Flow cytometry analysis of B lymphocyte cells in the blood (**F**) and tumor tissues (**G**) of mice. Representative images of stained samples and quantification of PCNA (**H**) and Ki67 (**I**) staining in tumor tissue from the *Csde1*-KO and *Csde1*-KO groups treated with CD20 (*n* = 4).

On the basis of the above results, we hypothesized that knocking out *Csde1* inhibited tumor development by increasing B-cell infiltration in the TME. To further validate this, we performed B-cell clearance in vivo by injecting a CD20 antibody (Fig. 7A). As shown in Fig. 7B–D, the tumor volume and weight were significantly reduced in the *Csde1*-knockout mice. However, upon B-cell depletion, the reduction in tumor volume and weight was markedly suppressed. These findings indicate that the immunoregulatory function of CSDE1 is mediated through B cells. During this period, mouse body weight was monitored, and neither *Csde1* knockout nor B-cell depletion affected body weight (Fig. 7E). Next, we evaluated the efficiency of B-cell depletion. As shown in Fig. 7F, G, the proportion of B cells in both blood and tumor tissues was significantly lower in the CD20-treated group than in the IgG control group. These results confirmed that CD20 treatment effectively depleted B cells in both the blood and tumor microenvironments. We subsequently performed immunohistochemical staining for the proliferation markers PCNA and Ki67 in tumor tissues. As shown in Fig. 7H, I, the expression levels of PCNA and Ki67 increased following CD20 treatment. These results indicate that B-cell depletion reversed the reduction in tumor stemness caused by CSDE1 knockout.

## Discussion

It has been reported that CSDE1 is correlated with different types of cancers, including melanoma, colorectal cancer, glioma, breast cancer, epithelial ovarian cancer, pheochromocytomas, paragangliomas, and hepatocellular carcinoma (HCC) [2, 3]. However, the mechanism by which CSDE1 plays a role in tumors is unclear. Most studies on the role of CSDE1 in tumors have focused mainly on its prognostic relevance and cell phenotype, without exploring its underlying mechanisms in depth [5, 6, 25, 26]. Only a few studies have reported that the biochemical mechanism of CSDE1 in melanoma and HCC is related to regulating the translation of the VIM and RAC1 [3, 4]. In this study, we demonstrated that CSDE1 promoted tumor progression by reshaping the tumor immune environment in a B-cell-dependent manner in NSCLC.

In the past, most studies on TIME focused primarily on tumor-infiltrating lymphocytes, particularly two major subsets of T cells: CD4^+^ T cells and CD8^+^ T cells [27, 28]. In the antitumor immune response, CD4^+^ T cells produce cytokines with chemotactic, proinflammatory, and immune-protective properties, while CD8^+^ T cells exert direct cytotoxic effects to eliminate tumor cells [18]. In recent years, significant progress has been made in understanding the role of tumor-infiltrating B cells (TIL-Bs), including both B cells and plasma cells, in the tumor immune response [20, 29]. B cells can recognize and present antigens, secrete antibodies, produce cytokines, generate immunological memory, and differentiate into plasma cells that produce IgG or IgA [30]. Our results revealed how the TIME and immune response are reshaped by CSDE1. We found that CSDE1 did not influence the levels of CD4^+^ T cells or CD8^+^ T cells but significantly increased the abundance of B cells and the levels of IgA, IgD, and IgG in the tumors of *Csde1*-KO mice. B cells are abundant in immune organs, particularly the spleen. Therefore, we analyzed the structure of the immune organs of the mice and the abundance of various immune cells in the spleen, which did not significantly change in the *Csde1*-KO mice. These results suggest that the increase in B-cell levels was specific to the TIME rather than occurring throughout the entire body.

B cells are the principal effectors of humoral immunity. A defining feature of adaptive immunity is the differentiation of B cells into antibody-secreting cells (ASCs), which produce large quantities of antibodies with or without T cell help and represent the terminal stage of B cell differentiation [31]. Immunoglobulin kappa C (Igkc) is highly expressed in ASCs and is widely used as an ASC biomarker. Igkc contributes to antibody diversity and immune function and is a key component of humoral immunity. High Igkc expression has been reported as an independent prognostic factor associated with improved survival in cancer patients [21, 32]. In our study, Igkc mRNA expression in B cells (Fig. 4C) and tissue levels of IgA, IgD, and IgG (Fig. 6H) were significantly increased in *Csde1*-KO tumors. These findings suggest that CSDE1 may promote tumor progression by suppressing B-cell-mediated humoral immunity through downregulation of Igkc mRNA. This hypothesis warrants further investigation.

Previous studies have identified a wide variety of surface molecules and diverse B-cell subsets distributed across multiple tissues and organs [33, 34]. In this study, we categorized B cells into nine subgroups, with memory B cells, tissue-resident B cells, Chl1^+^ B cells, and Vps37b^+^ cancer-immune B cells accounting for the highest proportions. Memory B cells can originate from B-cell differentiation and respond rapidly to antigen rechallenge [35]. Memory B cells and tissue-resident B cells have also been reported to be enriched in other tumors, such as pancreatic cancer [36]. Chl1^+^ B cells and Vps37b^+^ cancer-immune B cells were newly identified in our study and were named on the basis of the top marker gene expressed in these subgroups. However, the specific functions of these newly discovered B-cell subgroups remain unclear and require further investigation.

To further evaluate the inhibitory effect of B cells on tumor progression in *Csde1*-KO mice, a CD20 antibody was administered to deplete B cells. This treatment effectively rescued the inhibitory effect of *Csde1* knockout on tumor progression. However, no significant difference was detected in tumor burden between *Csde1*-WT mice with or without CD20 antibody treatment. This could be attributed to the maturity of B cells infiltrating the tumor tissue, as the CD20 antibody can only temporarily deplete mature B cells that express CD20.

In conclusion, this study is the first to uncover the role of CSDE1 in NSCLC. Our findings reveal that *Csde1* knockout significantly suppresses NSCLC progression by reshaping the TIME in a B-cell-dependent manner. These results highlight CSDE1 as a promising potential target for immunotherapy.

## Supplementary information

Supplementary file

## Data Availability

The data will be made available upon request.
