# Supplementary material for: CSDE1 depletion inhibits tumor progression through enhancing B-cell infiltration in NSCLC
Source: Cell Death Dis. 2025 Dec 6;17(1):140. doi: 10.1038/s41419-025-08282-9 (PMC12847937; doi:10.1038/s41419-025-08282-9)
Supplement: Supplementary file 1 — Supplementary file [file 41419_2025_8282_MOESM1_ESM.pdf]

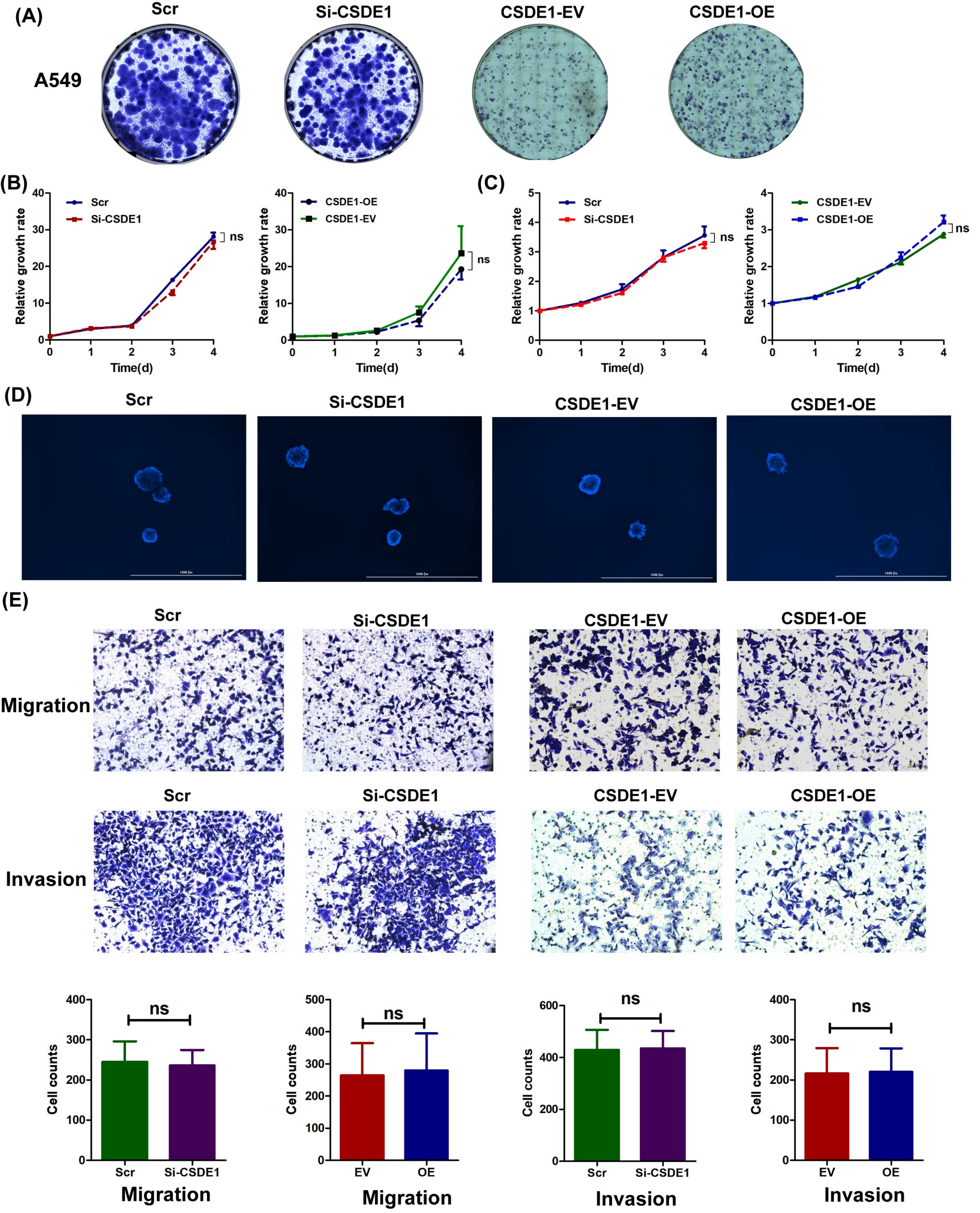

(A)
Scr
Si-CSDE1
CSDE1-EV
CSDE1-OE
A549
(B)
Scr
Si-CSDE1
CSDE1-OE
CSDE1-EV
Relative growth rate
Time(d)
ns
(C)
Scr
Si-CSDE1
CSDE1-EV
CSDE1-OE
Relative growth rate
Time(d)
ns
(D)
Scr
Si-CSDE1
CSDE1-EV
CSDE1-OE
(E)
Scr
Si-CSDE1
CSDE1-EV
CSDE1-OE
Migration
Invasion
Cell counts
ns
Scr
Si-CSDE1
EV
OE
Migration
Migration
Invasion
Invasion

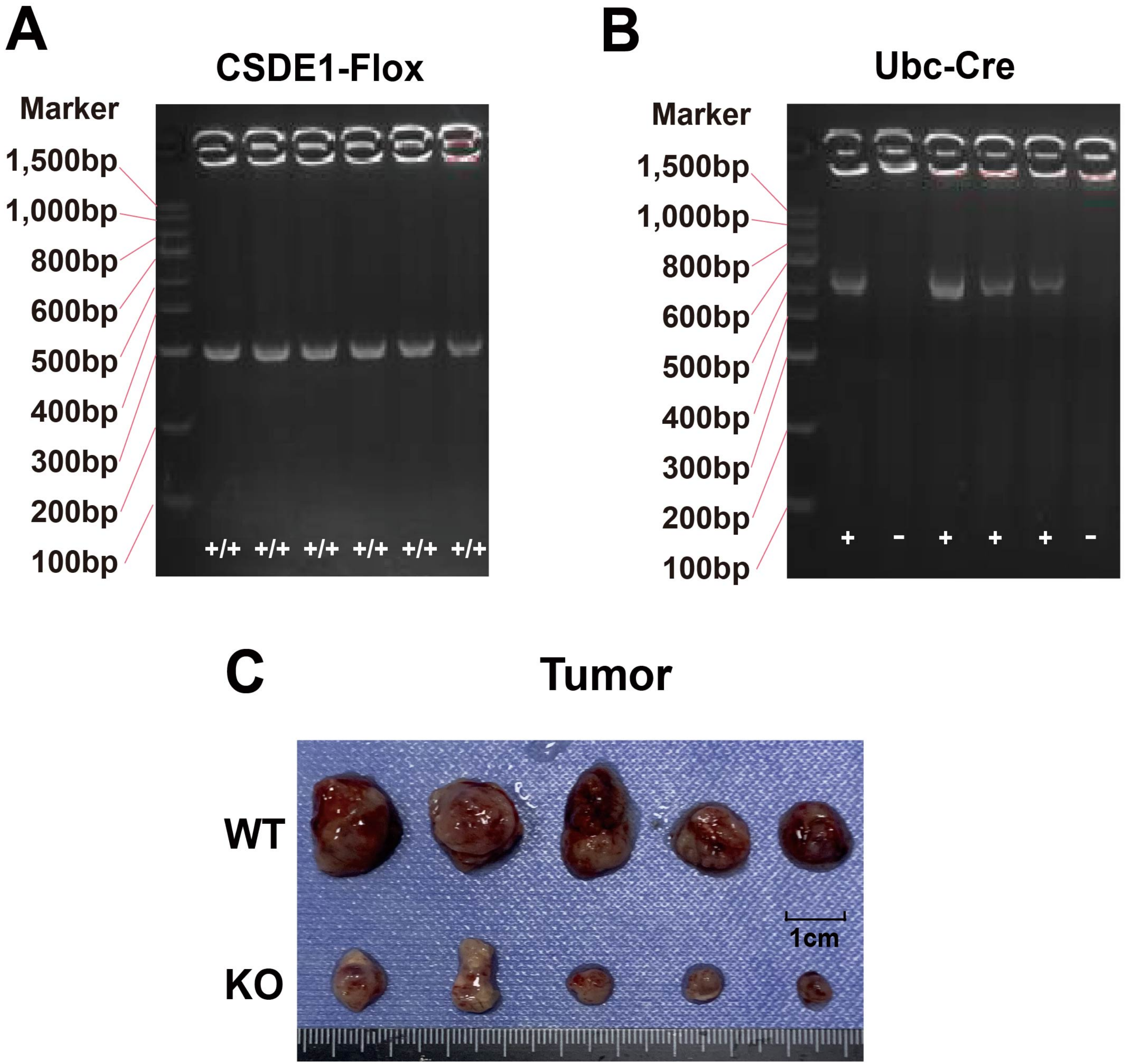
A
CSDE1-Flox
Marker
1,500bp
1,000bp
800bp
600bp
500bp
400bp
300bp
200bp
100bp
+/+ +/+ +/+ +/+ +/+ +/+
B
Ubc-Cre
Marker
1,500bp
1,000bp
800bp
600bp
500bp
400bp
300bp
200bp
100bp
+ - + + + -
C
Tumor
WT
KO
1cm

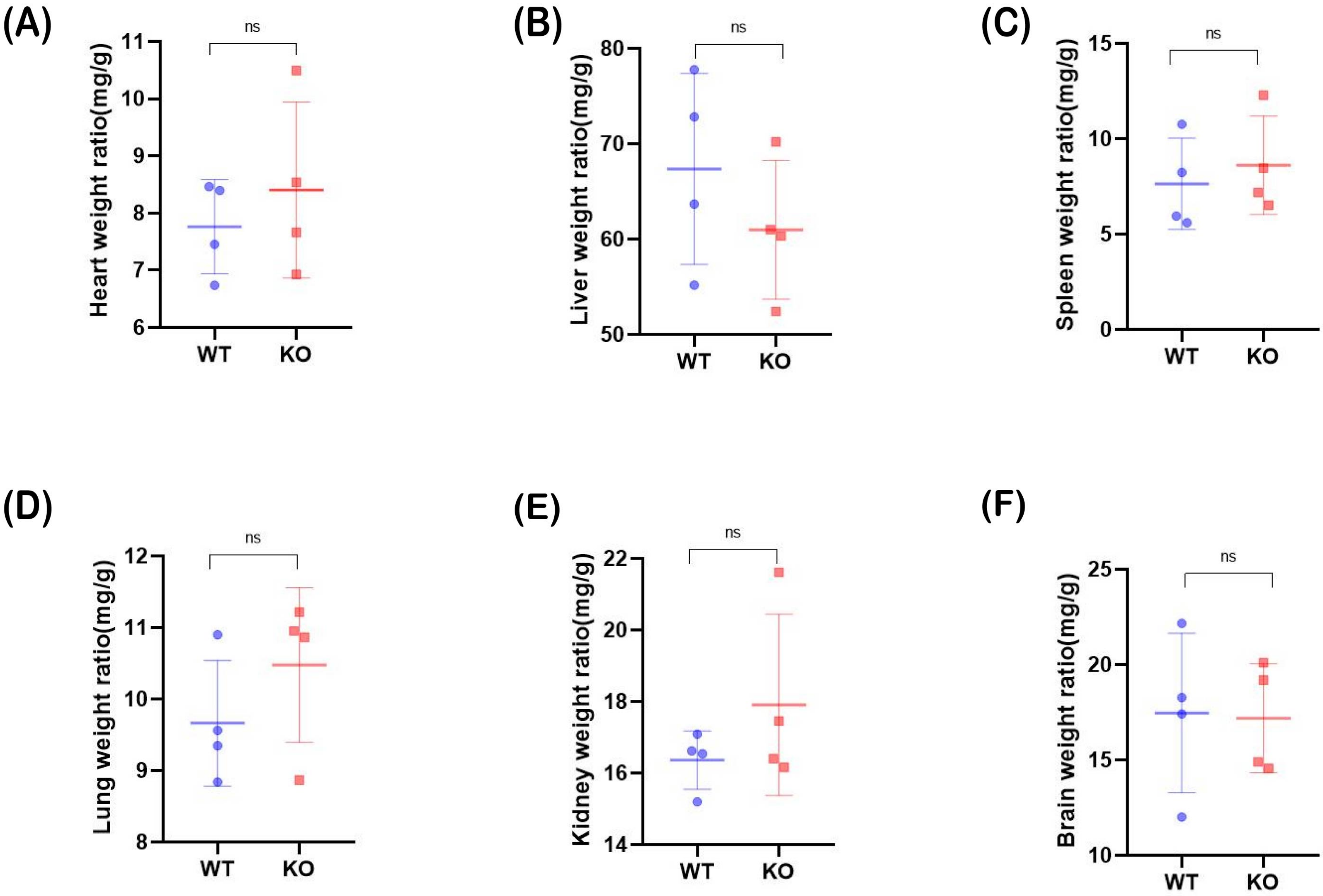
(A)
Heart weight ratio(mg/g)
ns
WT
KO
(B)
Liver weight ratio(mg/g)
ns
WT
KO
(C)
Spleen weight ratio(mg/g)
ns
WT
KO
(D)
Lung weight ratio(mg/g)
ns
WT
KO
(E)
Kidney weight ratio(mg/g)
ns
WT
KO
(F)
Brain weight ratio(mg/g)
ns
WT
KO

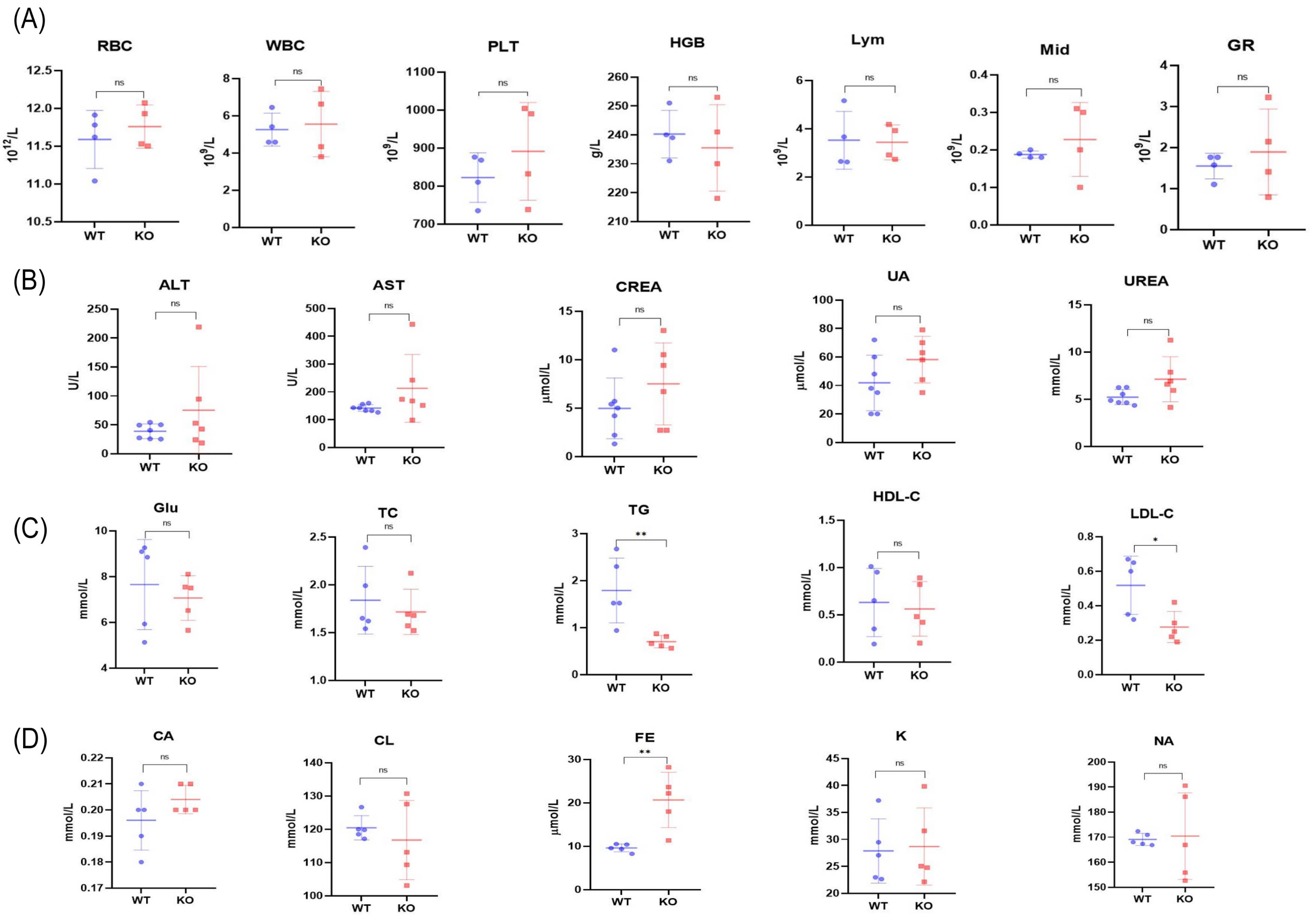
(A)
RBC
WBC
PLT
HGB
Lym
Mid
GR
(B)
ALT
AST
CREA
UA
UREA
(C)
Glu
TC
TG
HDL-C
LDL-C
(D)
CA
CL
FE
K
NA
WT
KO
ns
**
*
mmol/L
μmol/L
U/L
g/L

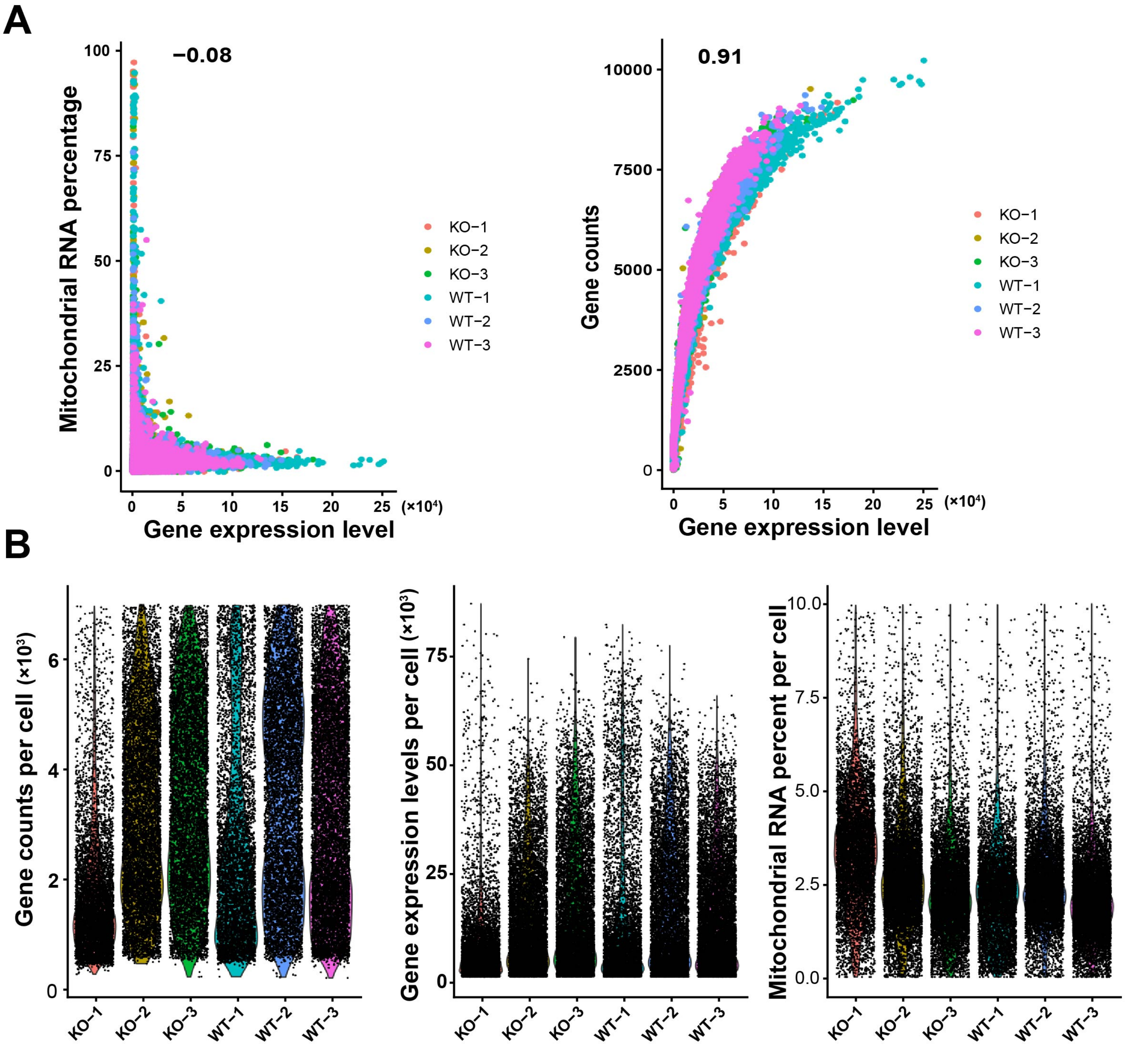
A
−0.08
Mitochondrial RNA percentage
Gene expression level
(×10^4)
KO−1
KO−2
KO−3
WT−1
WT−2
WT−3
0.91
Gene counts
B
Gene counts per cell (×10^3)
Gene expression levels per cell (×10^3)
Mitochondrial RNA percent per cell

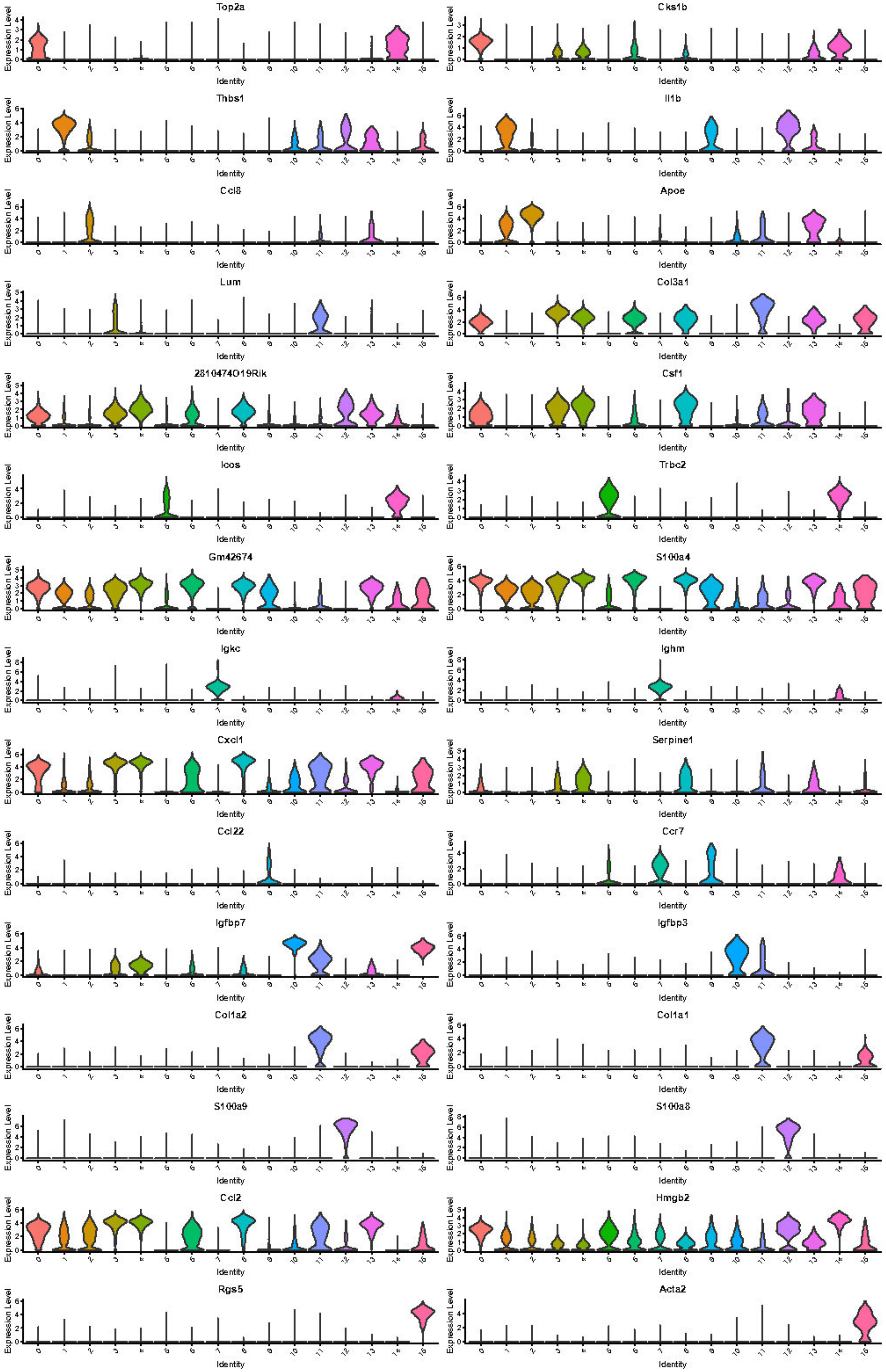
Top2a
Cks1b
Thbs1
Il1b
Ccl8
Apoe
Lum
Col3a1
2810474O19Rik
Csf1
Icos
Trbc2
Gm42674
S100a4
Igkc
Ighm
Cxcl1
Serpine1
Ccl22
Ccr7
Igfbp7
Igfbp3
Col1a2
Col1a1
S100a9
S100a8
Ccl2
Hmgb2
Rgs5
Acta2
Expression Level
Identity

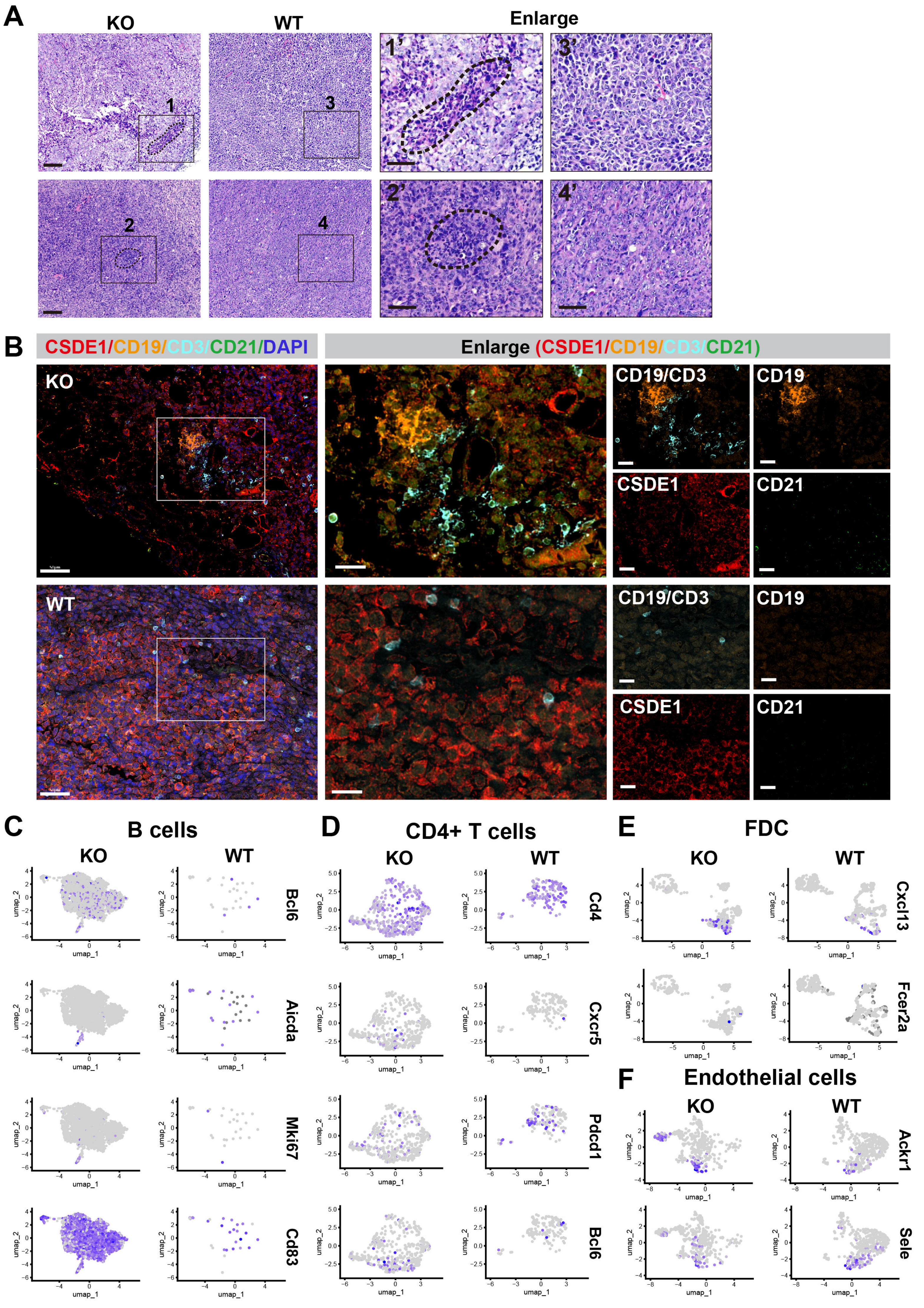

A
KO
WT
Enlarge
1
2
3
4
1'
2'
3'
4'
B
CSDE1/CD19/CD3/CD21/DAPI
Enlarge (CSDE1/CD19/CD3/CD21)
KO
WT
CD19/CD3
CD19
CSDE1
CD21
C
B cells
Bcl6
Aicda
Mki67
Cd83
D
CD4+ T cells
Cd4
Cxcr5
Pdcd1
Bcl6
E
FDC
Cxcl13
Fcer2a
F
Endothelial cells
Ackr1
Sele
umap_1
umap_2

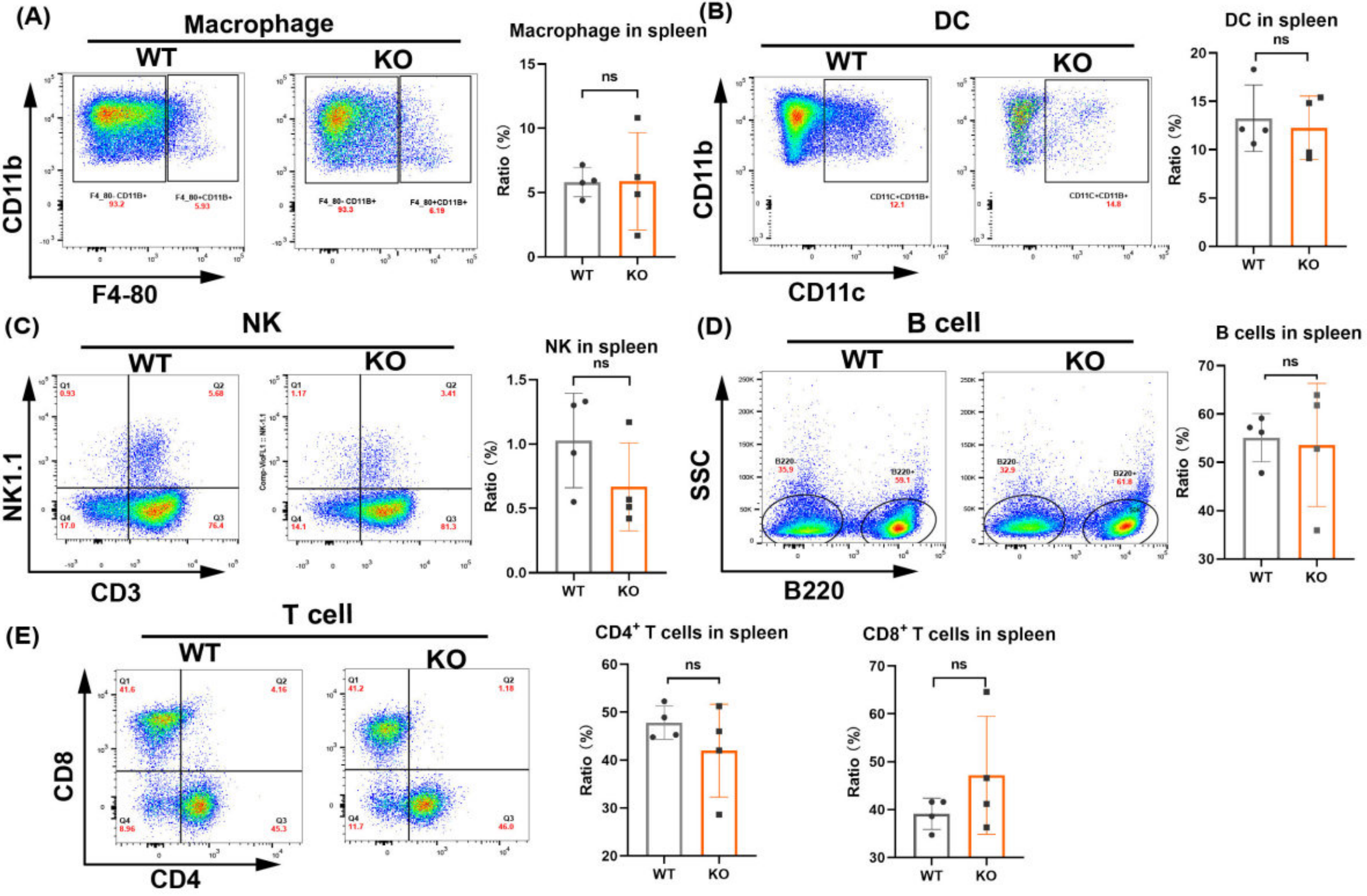

(A)
Macrophage
WT
KO
CD11b
F4-80
F4_80- CD11B+
93.2
F4_80+CD11B+
5.93
93.3
6.19
Macrophage in spleen
ns
Ratio（%）
WT
KO
(B)
DC
WT
KO
CD11b
CD11c
CD11C+CD11B+
12.1
14.8
DC in spleen
ns
Ratio（%）
(C)
NK
WT
KO
NK1.1
CD3
Q1
0.93
Q2
5.68
Q4
17.0
Q3
76.4
1.17
3.41
14.1
81.3
NK in spleen
ns
Ratio（%）
(D)
B cell
WT
KO
SSC
B220
B220-
35.9
B220+
59.1
32.9
61.8
B cells in spleen
ns
Ratio（%）
(E)
T cell
WT
KO
CD8
CD4
Q1
41.6
Q2
4.16
Q4
8.96
Q3
45.3
41.2
1.18
11.7
46.0
CD4+ T cells in spleen
CD8+ T cells in spleen
ns
Ratio（%）

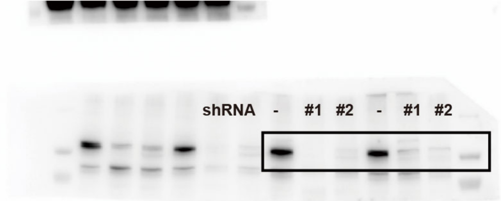

CSDE1

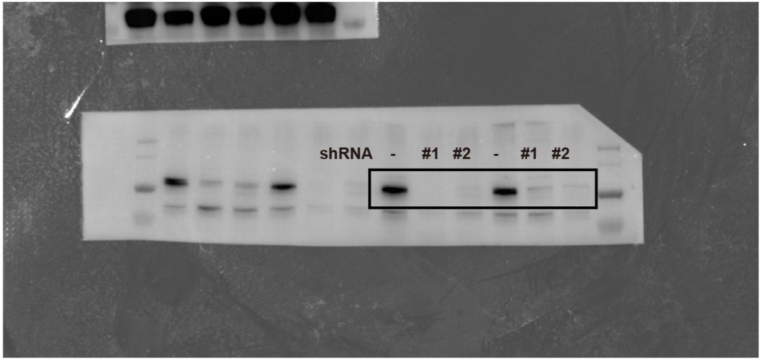

CSDE1

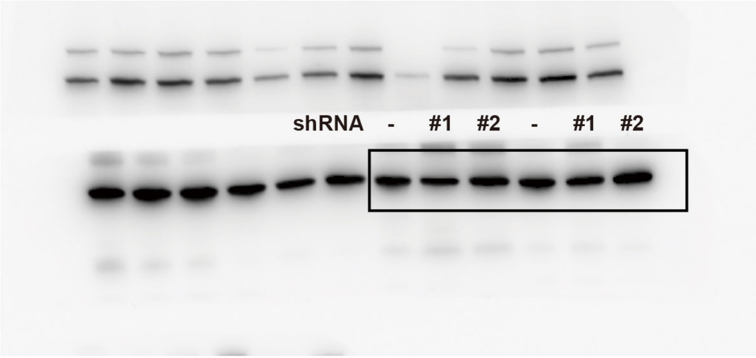

β-Actin

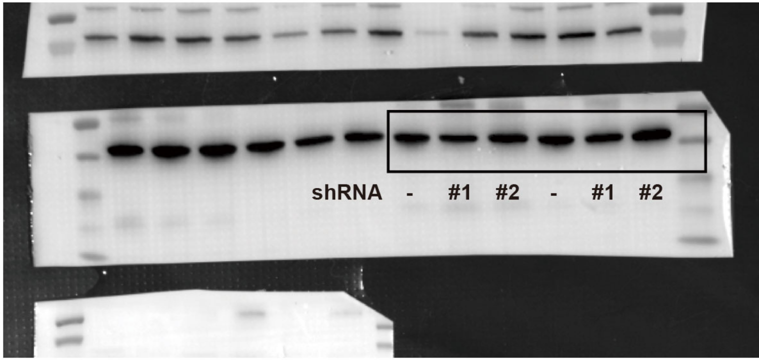

β-Actin

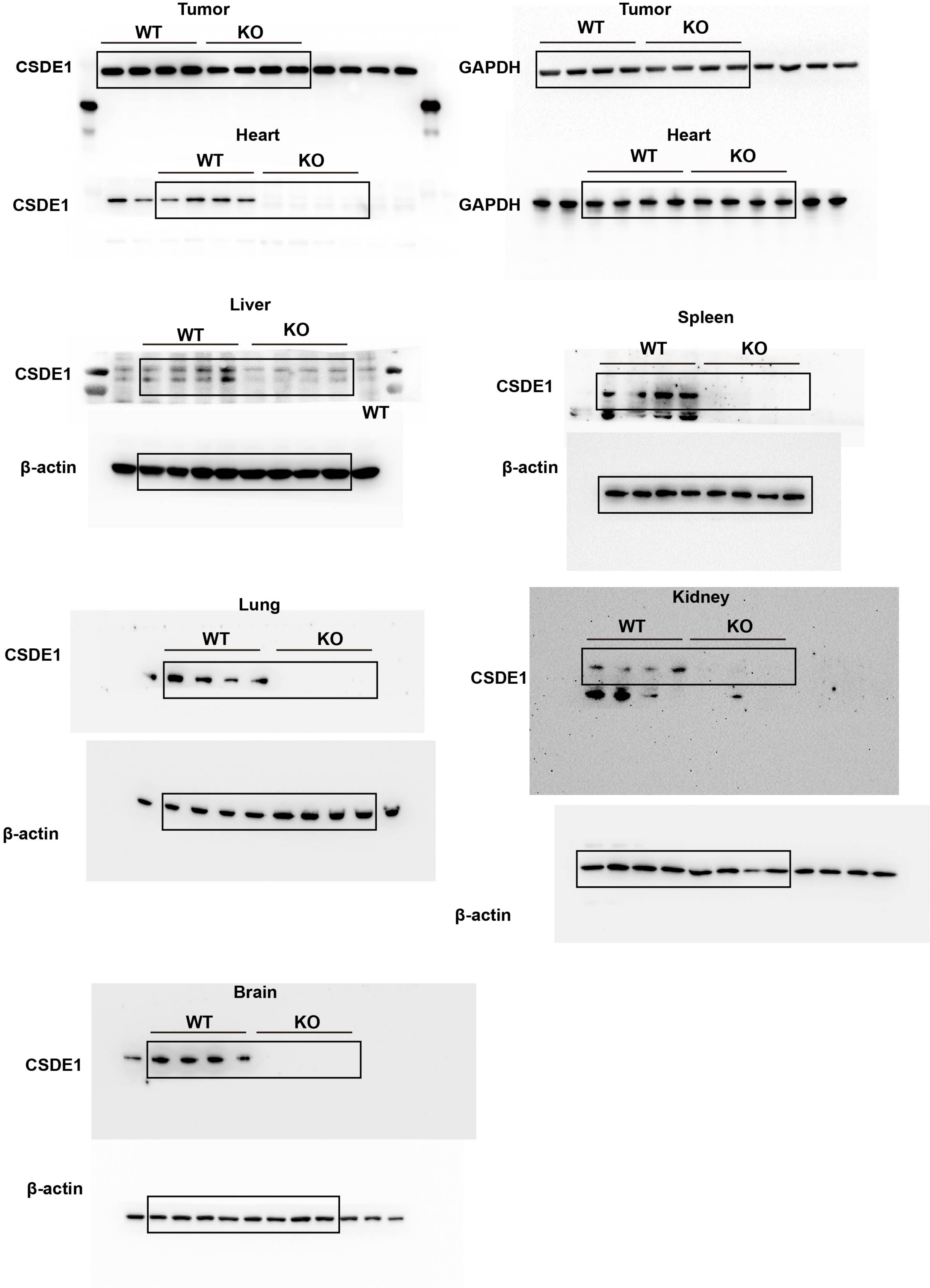

Tumor
WT
KO
CSDE1
Tumor
WT
KO
GAPDH
Heart
WT
KO
CSDE1
Heart
WT
KO
GAPDH
Liver
WT
KO
CSDE1
WT
β-actin
Spleen
WT
KO
CSDE1
β-actin
Lung
WT
KO
CSDE1
β-actin
Kidney
WT
KO
CSDE1
β-actin
Brain
WT
KO
CSDE1
β-actin

Supplementary figure legends

Figure S1. CSDE1 had no significant effect on tumor progression in A549 cells. (A) Colony formation assays of A549 cells with high or low CSDE1 expression. (B-C) Proliferation of A549 cells with high or low expression of CSDE1, as detected by a CCK8 assay. (D) Fluorescence image of DAPI-stained A549 cells cultured in matrix gel on the 4th day with high or low expression of CSDE1. (E) Transwell migration and invasion assays in A549 cells with high or low expression of CSDE1.

Figure S2. Successful construction of a *Csde1*-KO mouse model with tumor bearing ability. (A-B) Gel electrophoresis of the toe DNA from *Csde1*$^{\mathrm{Flox/Flox}}$-Cre(-) and *Csde1*$^{\mathrm{Flox/Flox}}$-Cre(+) mice. (C) Representative morphological images of tumors (n=5).

Figure S3. Organ-to-body weight ratios of the mice (n=4). (A) Heart-to-body weight ratio. (B) Liver-to-body weight ratio. (C) The spleen-to-body weight ratio. (D) The lung-to-body weight ratio. (E) The kidney-to-body weight ratio. (F) The brain-to-body weight ratio.

Figure S4. Comparison of blood biochemical indices between the *Csde1*-WT and *Csde1*-KO groups. (A) Routine blood indices (n=4). (B) Representative indices of liver and kidney function (*Csde1*-WT group: n=7; *Csde1*-KO group: n=6). (C) Representative indices of blood glucose and blood lipids. (D) Representative indices of the serum electrolytes test.

Figure S5. scRNA-seq data quality control chart. (A) Scatter plots of Pearson correlation analysis for the percentage of mitochondrial RNA (left), gene count, and gene expression level (right) across different samples. Left: the relationship between the gene expression level (x-axis) and the percentage of mitochondrial RNA (y-axis); right: the relationship between the gene expression level (x-axis) and the gene count (y-axis). Different colors represent samples, the numbers represent Pearson correlation coefficients, and each point represents a cell. (B) Quality control violin chart. Left: gene

counts per cell; middle: gene expression levels per cell; right: the proportion of mitochondrial RNA per cell.

Figure S6. Single-cell sequencing marker gene expression violin diagram. The horizontal axis of each graph represents different cell populations, the vertical axis indicates the expression level of marker genes, and the varying colors denote distinct clusters.

Figure S7. The effect of *Csde1* knockout on TLS maturation. (A) Representative images of HE stained samples in tumor tissue from the *Csde1*-WT and *Csde1*-KO groups. Scale bars: 100 μm (left), 50 μm (right). (B) Multiplex immunohistochemistry analysis of TLSs, including B cells (CD19), FDCs (CD21), T cells (CD3), in tumor tissue from the *Csde1*-WT and *Csde1*-KO groups. Representative images are shown. Scale bars: 50 μm (left), 20 μm (right). (C-F) The expression of biomarkers in B cells, $CD4^+$ T cells, FDC and endothelial cells in tumor tissues from *Csde1*-WT and *Csde1*-KO mice based on the single-cell RNA sequencing results.

Figure S8. The abundance of immune cells in the spleens of *Csde1*-knockout mice. (A-E) Flow cytometry analysis of B lymphocytes, DCs, NK cells, macrophages and T lymphocytes from tumor tissues. Representative images and quantification are shown.

Figure S9. The original WB images of Figure 1L which indicates the knockdown efficiency of CSDE1 in tumors from nude mice transplanted with H1299 cells.

Figure S10. The original WB images of Figure 2D which indicates the expression of CSDE1 in tumors and major organs, including the heart, liver, spleen, lung, kidney and brain.
